# Effects of Different Carbohydrate Levels in Diets on Growth Performance and Muscle Nutritive Value of Ying Carp and Scattered-Scaled Mirror Carp (*Cyprinus carpio*)

**DOI:** 10.1155/anu/9966429

**Published:** 2025-01-28

**Authors:** Pengfei Xiao, Yunya Wu, Hang Sha, Xiangzhong Luo, Guiwei Zou, Hongwei Liang

**Affiliations:** ^1^College of Fisheries and Life Science, Shanghai Ocean University, Shanghai 201306, China; ^2^Yangtze River Fisheries Research Institute, Chinese Academy of Fisheries Science, Wuhan 430223, China

## Abstract

This experiment aimed to assess the dietary adaptation and utilization of high carbohydrate diets to Ying carp and scattered-scaled mirror carp (*Cyprinus carpio*), focusing on growth performance, muscle nutritive value, and nutrient metabolism. Ying carp (4.5 ± 0.2 g) and scattered-scaled mirror carp (5.01 ± 0.2 g) were fed isonitrogenous diets containing 20%, 30%, and 40% carbohydrates for 8 weeks; the nitrogen content of the three feeds was (5.12% ± 0.03%). After the trail, the final body weight, feed efficiency, and specific growth rate of both carp varieties were analyzed. Results showed that the final body weight, feed efficiency, and specific growth rate of both carp varieties were significantly higher at the 30% carbohydrate level compared to 20% and 40%, indicating improved growth performance (*p* < 0.05). Crude protein content in whole fish composition was significantly higher at the 30% carbohydrate level compared to the other two levels (*p* < 0.05) for both varieties. However, excessive carbohydrate intake (40%) led to pronounced liver fat deposition in both varieties, with scattered-scaled mirror carp showing less severe deposition than Ying carp. As the carbohydrate levels in the feed increased, the essential amino acid (EAA) and total amino acid (TAA) content in the muscle of both carp varieties significantly increased, while the content of unsaturated fatty acids in the muscle significantly decreased (*p* < 0.05). Gene expression analysis revealed enhanced glycolytic activity (*pk1*) and inhibited gluconeogenesis (*g6p* and *pepck*) in the liver with higher carbohydrate levels. In muscle tissue, high carbohydrate diets reduced expression levels of genes involved in polyunsaturated fatty acid (PUFA) synthesis (*elovl5*, *elovl6*, and *rxrgb*). The two carp varieties exhibited distinct adaptations to varying dietary carbohydrate levels. While a 30% carbohydrate diet enhanced the growth performance of both varieties, scattered-scaled mirror carp demonstrated higher growth efficiency, whereas Ying carp excelled in maintaining muscle nutrient quality, particularly regarding PUFAs and amino acid composition. These findings suggest that carbohydrate levels in feed should be optimized based on the specific goals of aquaculture, whether prioritizing rapid growth or improved muscle nutrition. Moreover, variations in the expression of genes related to carbohydrate metabolism between the two varieties influenced their metabolic responses, offering insights for designing variety-specific feeding strategies to support sustainable aquaculture practices.

## 1. Introduction

In recent years, global fish consumption has significantly increased, with aquaculture projected to contribute 49% of total production by 2020 [1]. This expansion highlighted the critical importance of aquaculture in ensuring global food security, particularly in light of the rising demand for food. However, challenges such as rising costs of key aquafeed ingredients like fishmeal and soybean meal constrained profitability, hindering the sector's expansion [2–4]. Addressing these challenges was crucial, with current research focusing on identifying alternative protein sources and cost-effective nonprotein energy sources [5].

Carbohydrates constituted a crucial nonprotein energy source in fish nutrition and were highly valued for their cost-effectiveness in aquafeed formulations [6]. In fish, carbohydrates were primarily stored as glycogen in the liver and muscles, serving as a readily available energy reserve. These stored carbohydrates undergo various metabolic processes such as glycolysis, the tricarboxylic acid cycle, the pentose phosphate pathway, glycogen synthesis, and gluconeogenesis to meet the organism's energy demands [7]. Optimal levels of dietary carbohydrates enhanced fish growth performance [8]. However, excessive carbohydrate intake leads to growth inhibition and metabolic disorders [9–11]. Generally, omnivorous and herbivorous fish exhibit higher carbohydrate utilization compared to carnivorous species [12]. Herbivorous fish adapted to higher dietary starch levels by upregulating glycolytic enzyme activity with continuous high-starch feedings [13]. In contrast, carnivorous fish had limited enzymatic regulation capacity, which hindered their ability to efficiently utilize dietary starch. For instance, excessive starch feeding in a large yellow croaker (*Larimichthys crocea*) was reported to decrease glucokinase activity, thereby impairing starch utilization [14, 15]. Furthermore, excessive dietary carbohydrates have been linked to reduced growth rates, lower feed conversion efficiencies, decreased protein utilization, and the development of fatty liver syndrome, compromising liver function [16].

The carbohydrate content in fish feed significantly influenced the nutritional quality of muscle tissue. Research on blunt snout bream (*Megalobrama amblycephala*) demonstrated that higher dietary carbohydrate levels notably alter the fatty acid composition of muscle, increasing monounsaturated fatty acids (MUFAs) while decreasing polyunsaturated fatty acids (PUFAs) content [9]. Additionally, diets rich in carbohydrates have been found to enhance water retention, decrease muscle hardness, and improved flavor in tilapia (*Oreochromis mossambicus*), though they simultaneously diminish the nutritional value of the amino acid profile [17].

The common carp (*Cyprinus carpio*) was renowned for its adaptability to diverse environments and food sources, making it economically significant as the third most widely cultured freshwater fish globally [9, 18]. Ying carp (*a hybrid of common carp*, *crucian carp*, *and mirror carp*) and scattered-scaled mirror carp (*C. carpio* L.) were two important varieties of carp cultured species in China. The Ying carp was developed by crossing female mirror carp with male trans-nucleus fish of the common carp and F_2_ generation crucian carp at the Yangtze River Fisheries Research Institute of the Chinese Academy of Fisheries Research. The Ying carp used in this study was from the F_5_ generation, selected through artificial breeding. Carp is an omnivorous fish with a high capacity to metabolize carbohydrates [19]. Differences in feed carbohydrate requirements of different carp varieties; for instance, Huanghe carp thrives on a 30% carbohydrate diet, while Jian carp achieves optimal growth with a 38% carbohydrate diet [20, 21]. However, there was limited research on Ying carp and scattered-scaled mirror carp when fed high-carbohydrate diets.

The research investigated growth performance, whole-body composition, muscle nutritional quality, and the impact of varying carbohydrate levels on liver function in both carp varieties. Additionally, the study analyzed changes in the expression patterns of hepatic glucose metabolism-related genes and muscle unsaturated fatty acid-related genes to comprehensively assess their growth performance, carbohydrate utilization capacity, and muscle nutritional quality. This research aims to establish a theoretical framework for understanding the interplay between carbohydrate metabolism, growth dynamics, and muscle nutritional characteristics in carp.

## 2. Materials and Methods

### 2.1. Experimental Feed Formulation and Feed Preparation

Three experimental feeds were prepared with varying carbohydrate levels of 20%, 30%, and 40%, alongside a fixed protein content of 22%. The formulations and chemical compositions are detailed in Table 1. The primary protein sources included fishmeal, soybean meal, and casein, while fish oil and soybean oil served as the main fat sources. Corn starch was utilized as the main carbohydrate component. All ingredients were initially sieved through a 40-mesh sieve. Subsequently, the mixture was extruded using a single screw extruder and air-dried until achieving a moisture content below 10%. The extruded material was then crushed into pellets with a diameter of 2.0 mm using a feed mill. These pellets were further dried at 60°C and subsequently stored at −20°C until use.

### 2.2. The Feeding Trial Management

Experimental YC and SSC, bred at Yaowan Experiment Farm, Yangtze River Fisheries Research Institute, Chinese Academy of Fisheries Sciences (Jingzhou, Hubei, China), were adapted to commercial feed for 2 weeks before a 24-h fasting period preceding the growth experiment. The initial weight of carp was (4.5 ± 0.2 g), and the initial weight of the loose-scaled mirror carp was (5.01 ± 0.2 g), the initial body length was (2 ± 0.01 cm), comprised 270 individuals. Fish were randomly distributed into 18 culture buckets (500 L each), with 30 fish per bucket and three replicates per treatment group. They were fed twice daily (at 8:00 and 18:00) to satiation over 8 weeks. Daily feed intake per tank was recorded, followed by a 50% water change postfeeding. During the experimental period, dissolved oxygen was maintained at 5.5–6.5 mg/L, water temperature was maintained at about 26–28°C, and the photoperiod was 12 h light and 12 hr darkness.

### 2.3. Sample Collections

At the experiment's conclusion, each tank underwent a 24-h starvation period. Fish were individually weighed and measured for length to calculate weight gain rate, feed efficiency, and condition factor. For analysis, five fish per tank were randomly selected, anesthetized with MS-222 (Sigma–Aldrich, USA), and dissected to collect liver and muscle tissues. Samples were promptly frozen in liquid nitrogen and stored at −80°C for subsequent RNA isolation and histological analysis. Muscle tissues from the ipsilateral side of both carp varieties were also stored at −80°C until analysis.

### 2.4. Growth Calculation

The growth parameters and diet utilization were calculated according to the following formulas:

  $$\begin{array}{c} \text{Weight gain rate }\left(\text{WGR},\%/d\right)=\left(Wt-W0\right)\times 100/W0, \end{array}$$

  $$\begin{array}{c} \text{Specific growth rate (SGR, \%/d)}=\left(lnWt-lnW0\right)\times 100/t, \end{array}$$

  $$\begin{array}{c} \text{Condition factor }\left({\text{CF, g/cm}}^{3}\right)=\left(Wt/L^{3}\right)\times 100, \end{array}$$

  $$\begin{array}{c} \text{Feed efficiency (FE)}=100\times \left(Wt-W0\right)/F. \end{array}$$

*Wt* is the final body weight, *W*0 is the initial body weight (g), *L* is the body length (cm), *t* is the number of days (*d*), and *F* is the mass of dry feed used (g).

### 2.5. Nutritional Composition Analysis

All chemical analyses of feed and whole fish followed standard methods [24]. Moisture content was determined by drying samples to constant weight in a BAT240-LGF constant temperature oven (Wuhan Boantech Instrument Technology, China) at 105°C. Crude fat content was measured using an SZF-06A fat tester (Shanghai Precision Instrumentation Co., Ltd., China). Crude ash content was determined using a SX-4-10 muffle furnace (Tianjin Taiste Instrument Co., Ltd., China) at 550 ± 25°C for 12 h after burning. Crude protein content was analyzed using a Kjeldahl nitrogen tester SKD-200 (Shanghai Peio Analytical Instruments Co., Ltd., China).

For hydrolyzed amino acid analysis, 0.5 g of sample was placed in a headspace bottle; 2 mL of 6 mol/L hydrochloric acid solution was added, sealed, and hydrolyzed at 110°C for 24 h. The hydrolysate was filtered, evaporated to dryness in a rotary evaporator, redissolved, neutralized, lyophilized, redissolved again, filtered through a 0.22 μm microfilter, and analyzed using a liquid chromatograph tandem mass spectrometer 1260-6420A (Agilent Technologies Co., Ltd., USA) after plotting a standard curve for 20 amino acids.

For fatty acid analysis, 0.5 g of homogenized sample was placed in a 50 mL cuvette, and 10 mL of 8.3 mol/L hydrochloric acid solution was added. Hydrolysis was conducted at 80°C for 2 h until complete. After hydrolysis, 10 mL of 95% ethanol and 10 mL of mixed solvent (petroleum ether and ethyl ether, 1:1 *v:v*) were added, vortexed, and the supernatant extracted three times. The extract was concentrated at 40°C, followed by the addition of 2% sodium hydroxide methanol solution, and refluxed at 80°C until oil droplets disappeared. After cooling, 7 mL of 15% boron trifluoride methanol solution was added, refluxed for 2 min, and then processed for analysis using a GC-2010Pro gas chromatograph (Shimadzu Corporation, Japan) [25].

### 2.6. Total RNA Extraction and Real-Time Quantitative PCR (RT-qPCR)

Total RNA was extracted from carp liver and muscle using the TRIzol Reagent Kit (Thermo Fisher Scientific Inc., USA). RNA quality was assessed with a Nanodrop 2000 spectrophotometer (Thermo Fisher Scientific Inc., USA). Reverse transcription was performed using the PrimeScript TM RT Reagent Kit (Vazyme Biotech Co., Ltd., China) for RT-qPCR. The mRNA sequences of genes related to sugar metabolism and PUFA synthesis in carp were obtained from NCBI, and qPCR primers were designed using Primer 6.0 (Table 2). The amplification efficiency of all primers used in the qPCR experiments was tested and ranged between 90% and 110%. qPCR was conducted on a Bio-Rad CFX96 Real-Time PCR System (Bio-Rad Laboratories, Inc., USA) under the following cycling conditions: 95°C for 15 s, 60°C for 30 s, 72°C for 10 s, followed by 72°C for 20 s, for 40 cycles. Each gene was analyzed in three biological replicates, and expression values relative to *β-actin* were calculated using the 2^−∆∆Ct^ method.

### 2.7. Histological Analysis of the Liver and Fat Content Determination

Twelve liver samples from each group were fixed in 4% paraformaldehyde (PFA) at 4°C. Following dehydration in 30% sucrose, the samples were embedded in O.C.T. compound and cryosectioned. Six sections were stained with Oil Red O to visualize fat deposition, while the remaining six were stained with hematoxylin and eosin (H & E). Imaging was performed using a microscope equipped with a CCD (NIKON ECLIPSE CI; Nikon Corporation, Japan). The relative area of lipid droplets was analyzed using Image Pro Plus 6.0, with the average of six images calculated for each group. Data are presented from three independent replicates.

### 2.8. Statistical Analysis

All data were expressed as mean ± standard deviation (SD) and analyzed using SPSS statistical software (version 22.0; IBM, USA). Growth data, whole fish body composition, liver fat content, muscle composition, and muscle fatty acid and amino acid content were analyzed for group differences using one-way ANOVA followed by Duncan's multiple comparison method. Two-way ANOVA was used to determine the optimal level of carbohydrates in the diet and the degree of adaptation to carbohydrates in different varieties of carp, with Duncan's test used for post-hoc analysis of significant differences between groups. Expression levels of glucose metabolism-related genes and PUFA-related genes under different carbohydrate treatments were analyzed using independent-sample *t*-tests. Statistical significance was set at *p* < 0.05.

## 3. Result

### 3.1. Effects of Diets With Different Carbohydrate Levels on the Growth Performance of Two Varieties of Carp

After 8 weeks of feeding experiments, the growth metrics of the two carp varieties at different carbohydrate levels are presented in Table 3. The final body weight, weight gain rate, feed efficiency, and specific growth rate of both carp varieties initially increased and then decreased with rising carbohydrate levels. At the 30% carbohydrate level, the weight gain rate for both varieties was significantly higher compared to the 20% and 40% levels (*p* < 0.05). When the carbohydrate level was raised to 40%, both varieties exhibited a significant decrease in feed efficiency, and the specific growth rate of SSC also decreased significantly (*p* < 0.05).

In all three treatments, SSC had significantly higher weight gain rates than YC (*p* < 0.05). Feed efficiency in SSC was significantly higher than in YC at the 30% and 40% carbohydrate levels (*p* < 0.05). The condition factor of YC showed no significant change with varying carbohydrate levels, while the condition factor of SSC was significantly higher at 20% and 40% carbohydrate levels compared to 30% (*p* < 0.05).

Dietary carbohydrate levels significantly affected final body weight, weight gain rate, feed efficiency, and specific growth rate (*p* < 0.05). Breed had significant effects on weight gain rate, feed efficiency, and specific growth rate (*p* < 0.05). The interaction between strain and carbohydrate levels was significant for the condition factor (*p* < 0.05).

### 3.2. Body Composition

Moisture, crude fat, and crude ash contents in both carp varieties showed an initial decrease, followed by an increase with rising carbohydrate levels in the diet, whereas crude protein exhibited the opposite trend (Table 4). At the 30% carbohydrate level, moisture content was significantly lower, and crude protein content was significantly higher in both varieties compared to the 20% and 40% levels (*p* < 0.05). Crude lipid content in YC was significantly lower at the 30% carbohydrate level compared to the 20% and 40% levels (*p* < 0.05), and in SSC, it was significantly lower at the 30% level compared to the 20% level (*p* < 0.05).

YC had significantly lower moisture content than SSC across all treatments (*p* < 0.05). The crude lipid content of YC was significantly higher than that of SSC at the 20% and 40% carbohydrate levels (p < 0.05). The crude protein content of YC was significantly higher than that of SSC at the 30% and 40% carbohydrate levels (*P* < 0.05).

The interaction between carbohydrate levels and varieties on muscle crude lipid content was highly significant (*p* < 0.05). Carbohydrate levels in the feed significantly affected moisture, crude ash, and crude protein content in both carp varieties (*p* < 0.05).

### 3.3. Fat Deposition in the Liver of Two Carp Varieties Fed at Different Carbohydrate Levels

**Figure 1:**
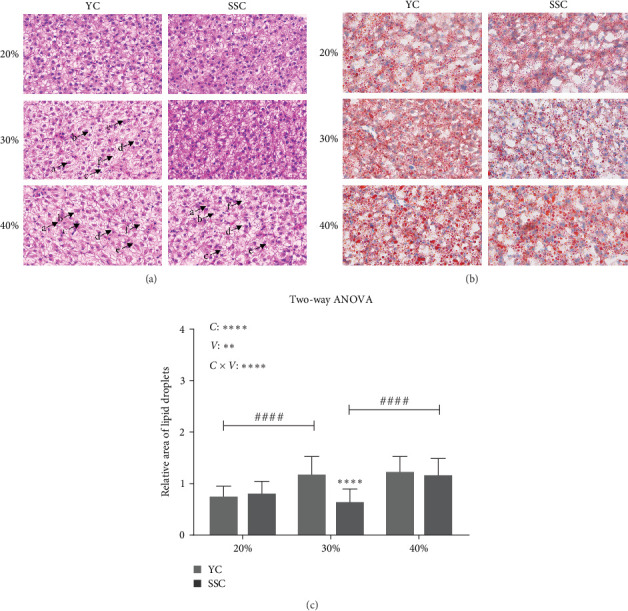
Fat deposition in the liver of two carp varieties fed at different carbohydrate levels. (A) Liver sections stained with H & E, 25 μm (400x); (a) hepatocyte nuclear displacement, (b) indistinct cell boundaries, (c) cytoplasmic fat droplet accumulation, (d) liver swelling, (e) cytoplasmic leakage, and (f) hepatocyte vacuolation; (B) liver sections stained with Oil Red, 25 μm (400x); (C) Relative lipid droplet area in liver. Each group of three repeats, totaling six groups *⁣*^*∗∗∗∗*^denotes significant differences between variety fed the same carbohydrate level diet (*p* < 0.0001), and ^####^denotes significant differences between the same breed fed different carbohydrate level diets (*p*  < 0.0001). A two-way ANOVA was used, *C* for carbohydrate level; *V* for variety, *⁣*^*∗∗*^(*p*  < 0.01), *⁣*^*∗∗∗∗*^(*p*  < 0.0001). H & E, hematoxylin and eosin.

H & E staining (Figure 1A) revealed normal hepatocyte arrangement, uniform cell size, distinct borders, and no damage in YC fed a 20% carbohydrate diet and SSC fed 20% and 30% carbohydrate diets. In contrast, YC fed 30% and 40% carbohydrate diets and SSC fed a 40% carbohydrate diet exhibited hepatocyte nuclear displacement, indistinct cell boundaries, cytoplasmic fat droplet accumulation, liver swelling, cytoplasmic leakage, and hepatocyte vacuolation.

Oil red O staining of liver sections (Figure 1B,C) showed a significant increase in liver lipid droplet content in YC with increasing dietary carbohydrate levels (*p* < 0.05). SSC displayed no significant increase in liver lipid droplets at 20% and 30% carbohydrate levels, but a significant increase was observed at the 40% carbohydrate level (*p* < 0.05). The relative area of liver lipid droplets was significantly higher in YC than in SSC at the 30% carbohydrate level (*p* < 0.05).

Both the level of dietary carbohydrates and the varieties of carp significantly affected liver fat deposition (*p*  < 0.05), with a highly significant interaction between carbohydrate levels and varieties on liver fat deposition (*p* < 0.05).

### 3.4. Amino Acid Composition in the Muscles of Two Varieties of Carp Fed With Different Carbohydrates

As shown in Table 5, the muscle content of total essential amino acids (EAAs), non-EAAs (NEAAs), delicious amino acids (DAAs), total semi-EAAs, and total amino acids (TAAs) in YC increased significantly with higher dietary carbohydrate levels (*p* < 0.05). In SSC, only the EAA content showed a significant increase, while other amino acid contents did not differ significantly.

The ratios EAA/TAA and EAA/NEAA were significantly higher in YC at the 30% carbohydrate level compared to the 20% and 40% levels (*p* < 0.05). In SSC, these ratios were significantly higher at the 30% level compared to the 20% level (*p* < 0.05). NEAA and DAA contents in SSC muscle were significantly higher than in YC at the 20% and 30% levels, with no significant difference at the 40% level (*p* < 0.05). EAA content in YC muscle was significantly lower than in SSC at the 20% and 40% levels (*p* < 0.05). At the 30% carbohydrate level, EAA/TAA and EAA/NEAA ratios were significantly higher in YC than in SSC (*p* < 0.05).

The carbohydrate level and varieties individually had significant effects on EAA, NEAA, DAA, and TAA contents (*p* < 0.05). Additionally, there were significant interaction effects between carbohydrate levels and varieties on EAA and TAA contents (*p* < 0.05).

### 3.5. Fatty Acid Composition in the Muscles of Two Varieties of Carp Fed With Different Carbohydrates

As shown in Table 6, the total saturated fatty acid content in the muscle of both carp varieties increased with higher carbohydrate levels in the feed (*p* < 0.05). The MUFA content in YC muscle decreased and then increased, while in SSC muscle, it increased and then decreased as carbohydrate levels rose (*p* < 0.05). PUFAs, including docosahexaenoic acid (DHA) and eicosapentaenoic acid (EPA), significantly decreased in the muscles of both carp varieties with higher dietary carbohydrate levels (*p* < 0.05). Across all three treatments, YC had significantly higher levels of PUFA, DHA, and EPA compared to SSC (*p* < 0.05). Both carbohydrate levels in the feed and the interaction of variety significantly affected the composition and content of PUFA in the muscle of both carp varieties (*p* < 0.05).

### 3.6. Effects of Feeding at Different Carbohydrate Levels on the Expression of Genes Related to Glucose Metabolism in the Liver

**Figure 2:**
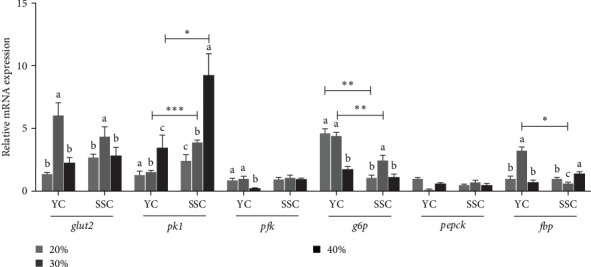
Relative mRNA expression levels of glucose metabolism-related genes in the livers of carp fed the test diets. Data are means ± SD (*n* = 3). The raw data related to this figure is provided in Supporting Information 1: Table S1. Values with different superscript letters in the same column indicate significant differences between different dietary treatments of the same variety at different carbohydrate levels (*p* < 0.05). *⁣*^*∗*^, *⁣*^*∗∗*^, and *⁣*^*∗∗∗*^ indicate significant differences (*p* < 0.05, *p* < 0.001, and *p* < 0.001) among different lines of the same carbohydrate level feed treatment. SD, standard deviation.

The effects of different carbohydrate levels in the diet on the expression levels of glucose metabolism-related genes in the liver of both carp varieties are illustrated in Figure 2. The expression of hepatic *glut2* in YC and SSC showed an initial increase followed by a decrease with increasing dietary carbohydrate levels. Notably, *glut2* expression was significantly higher in both carp varieties under the 30% carbohydrate diet compared to the other treatments (*p* < 0.05). Similarly, the expression levels of *pk1* in the liver significantly increased with rising dietary carbohydrate levels in both carp varieties (*p* < 0.05). Specifically, *pk1* expression was significantly higher in SSC than in YC at both the 30% and 40% carbohydrate levels (*p* < 0.05). Conversely, *g6p* exhibited a decreasing trend in YC as dietary carbohydrates increased, while SSC initially showed an increase followed by a decrease. The expression levels of *g6p* were significantly higher in YC compared to SSC at both the 20% and 30% carbohydrate levels (*p* < 0.05). No significant changes were observed in *pepck* and *pfk* expression levels across the different dietary treatments.

### 3.7. Effects of Feeding at Different Carbohydrate Levels on the Expression of Genes Related to Fatty Acid Synthesis in Muscle

**Figure 3:**
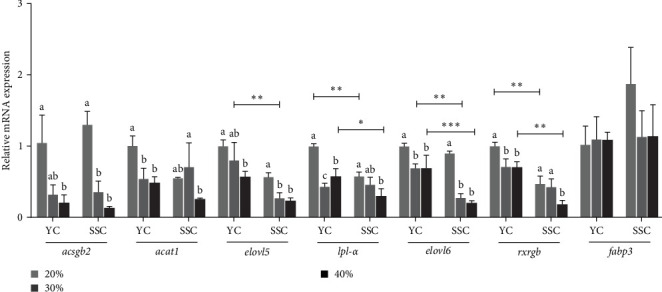
The relative mRNA expression levels of genes involved in the synthesis of polyunsaturated fatty acids in the muscles of fish-fed experimental diets. Data are means ± SD (*n* = 3). The raw data related to this figure is provided in Supporting Information 2: Table S2. The different superscript letters in the same column indicate significant differences in the responses of the same variety to different levels of dietary carbohydrates (*p* < 0.05). *⁣*^*∗*^, *⁣*^*∗∗*^, and *⁣*^*∗∗∗*^ indicate significant differences in the responses of different varieties to the same level of dietary carbohydrates (*p* < 0.05, *p* < 0.01, and *p* < 0.001). SD, standard deviation.

Figure 3 depicts the impact of varying carbohydrate levels in the diets on the expression levels of PUFAs in the muscles of both carp varieties. In YC and SSC, the expression levels of *acsbg2*, *elovl5*, *lpl-α*, *elovl6*, and *rxrgb* in muscle were significantly higher in the 20% treatment compared to the 40% treatment (*p* < 0.05). Moreover, the expression levels of *lpl-α* and *rxrgb* were significantly higher in the muscles of YC than in SSC at both the 20% and 40% treatments (*p* < 0.05). Additionally, at the 30% carbohydrate treatment, the expression levels of *elovl5* and *elovl6* were significantly higher in YC than in SSC (*p* < 0.05).

## 4. Discussion

### 4.1. Carbohydrates in Feed Promoted Growth Moderately but Excessive Levels Caused Severe Liver Fat Deposition

In this study, final body weight, weight gain, feed efficiency, and specific growth rate significantly increased in both carp varieties when fed a 30% carbohydrate diet compared to the 20% and 40% treatments. Similar findings have been reported for other fish species, such as grass carp (*Ctenopharyngodon idella*), silver Prussian carp (*Carassius auratus*), and blunt snout bream [26–28], suggesting that increasing dietary carbohydrate levels enhances carp growth performance. Two-way ANOVA analysis of the growth data indicated that varieties significantly influenced feed efficiency and weight gain rate in carp fed the 30% diet, with SSC exhibiting superior growth compared to YC. This difference may be attributed to enhanced feeding efficiency in both carp varieties with appropriately increased carbohydrate levels, resulting in increased feed intake and subsequent body weight [29]. However, when dietary carbohydrates were raised to 40%, final body weight, weight gain rate, feed efficiency, and specific growth rate significantly decreased in both carp varieties. This decrease is likely due to the limited capacity of fish to utilize carbohydrates effectively, leading to reduced growth performance [30, 31]. SSC fed the 40% carbohydrate diet showed a higher weight gain rate and specific growth rate compared to YC, indicating their better adaptation to high-carbohydrate nutritional environments.

The liver served as a crucial metabolic organ and the primary site for lipid storage in fish, making it sensitive to dietary carbohydrate levels, which could impact liver function and health [32, 33]. In this study, liver analysis revealed significant fat deposition, vacuolization of liver cells, and an increase in relative lipid droplet area in both carp varieties when fed 40% carbohydrate diets, similar to findings in European sea bass (*Dicentrarchus labrax*) [14]. Excessive lipid accumulation in fish liver could lead to liver damage and disruption of metabolic processes, thereby affecting overall fish health [34]. Both carp varieties exhibited pronounced fat accumulation at the 40% carbohydrate level, indicating compromised liver health unable to adapt to higher dietary carbohydrate levels. SSC showed lower liver fat deposition compared to YC, suggesting a greater adaptability of SSC to high-carbohydrate diets.

### 4.2. High Dietary Carbohydrates Promoted Carp Glycolysis, Inhibited Gluconeogenesis

Dietary carbohydrate levels influenced the expression of genes involved in glucose metabolism in fish [35]. Glut2, an important type of glucose transporter protein found in the liver, helped in the reabsorption of glucose in the intestine and kidney [36]. Studies in grass carp showed that glucose injection increases *glut2* expression levels [37]. In this experiment, *glut2* expression levels in the livers of both carp varieties were significantly higher in the 30% treatment compared to the other two treatments, indicating enhanced glucose uptake at moderate carbohydrate levels. Pk is one of the rate-limiting enzymes in glycolysis. In rainbow trout (*Oncorhynchus mykiss*), adding 20% carbohydrates to the diet can increase *pk* expression levels in the liver, promoting glycolysis [38]. In this experiment, feeding 40% carbohydrate diets significantly increased *pk* expression levels in the livers of both carp varieties, indicating that high carbohydrate levels can enhance glycolysis and improve carbohydrate utilization in carp.

G6p, an enzyme crucial for glucose synthesis in the gluconeogenic pathway [39], has been found to decrease in expression in response to 20% carbohydrate diets in studies on gilthead sea bream (*Sparus aurata* L.) and common carp [40, 41]. Similarly, in this experiment, *g6p* expression levels were significantly lower in the livers of YC fed the 40% treatment compared to the 20% and 30% treatments, while significantly higher in SSC fed the 30% treatment. This indicated that high carbohydrate diets can suppress gluconeogenesis, potentially by reducing the conversion of oxaloacetate and glucose-6-phosphate to phosphoenolpyruvate and glucose, respectively [42].

Furthermore, glycolysis-related gene expression levels were significantly lower in YC compared to SSC at the 30% and 40% carbohydrate levels, whereas gluconeogenesis-related gene expression was significantly higher in YC at the 20% and 30% levels. After feeding a high carbon water diet, the feeding environment is in an energy surplus; both carp are in a state of lower energy demand, with a reduced need for glycolysis, while the organism converts as much glucose as possible into glycogen stores [43]. These findings align with the superior growth performance and reduced liver fat deposition observed in SSC when maintained on a 30% carbohydrate diet. Overall, these results suggest that SSC may possess a more robust glycolytic capacity and lower gluconeogenic activity, enabling them to better utilize higher carbohydrate diets compared to YC.

### 4.3. Carbohydrates Raise Fish Protein, Increase Amino Acid Content and Decrease PUFA Content

During this study, the body composition of both carp varieties showed a pattern where moisture and crude lipids initially decreased and then increased, while crude protein levels increased and then decreased with rising dietary carbohydrate levels. Similar trends have been documented in studies on Nile tilapia (*Oreochromis niloticus*) and hybridized snakeheads (*Channa maculata♀ × Channa argus♂*) [19, 44], suggesting that optimal levels of dietary carbohydrates promote protein deposition and exhibit a significant protein-sparing effect. Additionally, there was a notable increase in crude lipids in the body composition of both carp varieties with increasing dietary carbohydrate levels, a phenomenon also observed in hibiscus carp and sea cucumber (*Apostichopus japonicus*) [45, 46]. This highlighted that excess carbohydrates added to the diet may contribute to lipid accumulation in fish muscle, potentially influencing the nutritional composition of the fish.

The content of DAAs and EAAs in muscle is crucial for assessing both the flavor and nutritional quality of fish [47]. In this study, the inclusion of higher levels of carbohydrates significantly increased EAAs in both carp varieties and elevated DAA levels, specifically in YC. These results align with findings from studies on Nile tilapia [17], indicating that increased dietary carbohydrates enhance the flavor of carp muscle. A high EAA/TAA ratio in fish muscle indicates a higher nutritional value for human consumption [48]. In accordance with the theoretical model proposed by the FAO/WHO, proteins were of better quality when EAAs comprise about 40% of TAAs, and the EAA/NEAA ratio exceeds 60%. In our study, both carp varieties achieved favorable values for EAA/TAA ratio and EAA/NEAA ratio across all treatments; however, excessively high carbohydrate diets led to a decrease in these ratios, potentially impacting muscle nutritional quality. Notably, YC exhibited lower levels of EAAs, DAAs, and TAAs compared to SSC yet demonstrated significantly higher EAA/TAA ratio and EAA/NEAA ratio ratios at the 30% treatment level. This suggested that SSC have higher levels of amino acids compared to YC. SSC preserves proteins by better-utilizing carbohydrates compared to YC; more proteins are used to accumulate amino acids [49]. However, in terms of muscle amino acid quality, YC had a more optimal proportion of amino acid composition.

The composition and content of fatty acids significantly influence the quality and flavor of fish muscle PUFAs, which were crucial for assessing nutritional value [50, 51]. DHA and EPA were essential PUFAs that played crucial roles in human health, particularly in cardiovascular health and cognitive function [52]. These fatty acids were abundant in fish and had been associated with reduced risk of cardiovascular disease and improved cognitive development, making them important components of a healthy diet [53]. In this study, we found that as the carbohydrate level in the feed increased, the contents of EPA, DHA, and PUFA in the muscles of both carp species gradually decreased. Therefore, we examined genes related to PUFA synthesis to understand their expression levels in muscle. Genes such as *elovl5* and *elovl6* were involved in PUFA elongation and DHA synthesis, while *rxrgb* supported fatty acid oxidation, promoting PUFA synthesis [54, 55]. Our findings revealed that high carbohydrate diets downregulated the expression of *elovl5*, *elovl6*, and *rxrgb* in the muscles of both carp varieties, suggesting an inhibitory effect on PUFA synthesis. Additionally, *acsgb2*, which converts linoleic acid to PUFAs [56], showed decreased expression levels in muscle, correlating with reduced PUFA content in both carp varieties' muscles under high carbohydrate diets. Similar trends have been observed in studies on Korean rockfish (*Sebastes schlegelii*) [57]. YC exhibited significantly higher PUFA content, including DHA and EPA, in muscle compared to SSC at 30% and 40% carbohydrate levels in the diet. Moreover, expression levels of *elovl6* and *rxrgb* were notably higher in YC, reflecting its superior ability to synthesize PUFAs in muscle, potentially linked to the higher crude lipid content in the whole-body composition of YC compared to SSC.

## 5. Conclusion

In conclusion, this study demonstrated that incorporating an appropriate amount of carbohydrates into carp diets enhances growth performance and acts as a protein economizer. This dietary adjustment also improves the amino acid composition of muscle but reduces the content of PUFAs, influencing both glucose metabolism and fatty acid synthesis. This study found that the PUFA content in the muscles of both carp varieties was optimal at a 20% carbohydrate level, while the amino acid composition was best at a 30% carbohydrate diet. Therefore, further investigation into the optimal carbohydrate-to-diet ratio is warranted to achieve the ideal balance of amino acids and fatty acids. The objective of this experiment was to assess the growth performance, carbohydrate utilization, and muscle nutritional quality of two carp varieties, providing a foundational understanding of the interplay between glucose metabolism, growth, and muscle nutritional quality in carp.

## Figures and Tables

**Table 1:** Formulation and proximate composition of the experimental diets (% dry basis).

Ingredient (%)	20%	30%	40%
Fishmeal	10	10	10
Soybean meal	16.4	16.4	16.4
Casein	20.5	20.5	20.5
Corn starch	20	30	40
Fish oil	2.3	2.3	2.3
Soybean oil	2.3	2.3	2.3
Carboxymethyl cellulose	3	3	3
Cellulose	20	10	0
Choline chloride	0.11	0.11	0.11
Vitamin premix^a^	0.39	0.39	0.39
Mineral premix^b^	5	5	5
Chemical composition (%)			
Moisture	10.67	11.11	13.94
Crude protein	31.98	31.9	32.20
Crude lipid	9.15	10.07	9.02
Ash	4.6	3.86	3.96
Digestibility carbohydrate^c^	23.6	33.06	40.08
Gross energyd (MJ kg^−1^)^d^	12.71	14.41	15.11

^a^Vitamin premix (g kg^−1^ premix): vitamin A 46,666,000 IU kg^−1^; vitamin D3 16,000,000 IU kg^−1^, vitamin K3 1.20, vitamin B1 1.60, vitamin B2 3.40, vitamin B6 3.60, vitamin B12 0.03, vitamin C 50.0, inositol l28.0, calcium pantothenate 6.0, folic acid 0.50, biotin 0.10, nicotinamide 8.0.

^b^Mineral premix (g kg^−1^ premix): KH_2_PO_4_ 135, NaCl 50, MgSO_4_·7H_2_O 150, KI 0.2, CoSO_4_.6H_2_O 0.3, CuSO_4_·5H_2_O 1.5, ZnSO_4_·7H_2_O 17.50, FeSO_4_·7H_2_O 125.0, MnSO_4_·H_2_O 8, Na_2_SeO_3_ 0.1.

^c^Digestibility carbohydrate = 100 − Crude protein − crude lipid − ash − fiber [22].

^d^Gross energy: Gross energy was calculated using energy equivalents 18.81, 35.57, and 14.59 kJ g^−1^ for protein, lipid, and digestible carbohydrate, respectively [23].

**Table 2:** Primer sequences for RT-qPCR.

Primer	Sequence (5′−3′)	Application
*rxrgb*-F	AATGAGGTGGAATCAACAAGCA	Fatty acid oxidation
*rxrgb*-R	GGCAGATCAGAGAAATGGGGTA	
*acat1*-F	TGTCTCAGGTTCCTTATGTTATGGC	Conversion of fatty acids to cholesterol
*acat*1-R	TGCTCACAGAAACCACTTCCTTG	
*elovl5*-F	CAGGATGATGAACGTACTTTGGTG	Polyunsaturated fatty acids prolong
*elovl5*-R	GGATGAAGCTGTTAAACGTGGC	
*lpl-α*-F	GAGTCAACAAAATTCGCACACG	fatty acid synthesis related
*lpl-α*-R	TTCAAAGCAGGCATAATGTAGGG	
*acsbg2*-F	CTCAAGTGTAACGTTGATGACAATG	Synthesis of EPA
*acsbg2*-R	AGATATGGAGAAGTCTCGTGGAAG	
*elovl6*-F	TAAGCATTGTGGGGCTCCTCTA	DHA synthesis
*elovl6*-R	CTCTCGTTTCACTCTCTTCTCGTCT	
*fabp3*-F	ACCAAGCCTACAACCATCATTT	DHA synthesis
*fabp3*-R	TCGTCTCTTTACCATCCCATTT	
*glut2-F*	CCAGCAGCCATCGCATTAGC	Glucose transport
*glut2-R*	GCACCATCTCAGCCTCTTCTTG	
*pk1-F*	AACGACGTGTGGGCCGAGGA	Glycolysis
*pk1-R*	TGGACGCCAGCCGGTCAGGA	
*pfk-F*	CACGTACAAGCTGTTAGCT	Glycolysis
*pfk-R*	TCGAAGCCATCATGGACGGT	
*g6p-F*	TGGTTGTTGCCGAGGCCTTCA	Gluconeogenesis
*g6p-R*	TGGGCTTTCTCCAGGGTCCACAGC	
*pepck-F*	GGTGCCCTCTTTGACCTGCCCAA	Gluconeogenesis
*pepck-R*	TCTGGCCTCCAGCGCCCTCA	
*fbp-F*	TGGTTCTCTCCACAGGCCAAGG	Gluconeogenesis
*fbp-R*	GGGCGAACTTCCATCCTCTGGGA	
*β-actin*-F	GATGATGAAATTGCCGCACTG	Housekeeping gene
*β-actin*-R	ACCAACCATGACACCCTGATGT	

Abbreviations: DHA, docosahexaenoic acid; EPA, eicosapentaenoic acid; RT-qPCR, real-time quantitative PCR.

**Table 3:** Growth performance of YC and SSC-fed diets with different carbohydrate levels.

Group	FBW (g)	WG (%)	FE	SGR (% d^−1^)	CF (g/cm^3^)
YC					
20%	14.43 ± 2.11^ab^	163.73 ± 3.32^d^	58.81 ± 5.73^b^	1.67 ± 0.27^c^	2.2 ± 0.1^b^
30%	15.04 ± 2.23^a^	190.51 ± 6.72^c^	59.52 ± 7.49^b^	1.74 ± 0.1^c^	2.3 ± 0.02^ab^
40%	13.99 ± 1.95^ab^	150.28 ± 5.9^e^	41.92 ± 8.7^c^	1.57 ± 0.11^c^	2.3 ± 0.17^ab^
SSC					
20%	14.25 ± 2.11^ab^	229.36 ± 4.84^b^	65.94 ± 3.99^ab^	2.08 ± 0.09^ab^	2.45 ± 0.04^a^
30%	15.51 ± 2.18^a^	259.28 ± 7.85^a^	71.62 ± 6^a^	2.2 ± 0.19^a^	1.95 ± 0.14^c^
40%	13.36 ± 1.83^b^	199.58 ± 7.33^c^	55.8 ± 3.96^b^	1.86 ± 0.09^bc^	2.36 ± 0.04^ab^
Two-way ANOVA					
Carbohydrate	0.0198	0.0104	0.0029	0.0398	0.0946
Variety	0.7507	0.0015	0.0353	0.0111	0.8284
Carbohydrate × variety	0.5436	0.0628	0.234	0.4623	0.0245

*Note*: Data are means ± SD (*n* = 3). Values with different superscript letters in the same column show significant differences (*p*  < 0.05).

Abbreviations: CF, condition factor; FBW, final body weight; FE, feed efficiency; SGR, specific growth rate; WG, weight gain rate.

**Table 4:** Body composition of two varieties of carp fed with different carbohydrate level diets for 8 weeks.

Body (%)	Moisture	Crude lipid	Crude ash	Crude protein
YC				
20%	77.71 ± 0.35^b^	2.55 ± 0.05^a^	1.30 ± 0.04^ab^	17.68 ± 0.24^cd^
30%	76.55 ± 0.33^c^	1.54 ± 0.05^e^	1.22 ± 0.08^b^	20.00 ± 0.41^a^
40%	77.90 ± 0.39^b^	2.07 ± 0.04^b^	1.28 ± 0.05^ab^	18.15 ± 0.36^bc^
SSC				
20%	79.01 ± 0.36^a^	1.67 ± 0.04^c^	1.24 ± 0.03^b^	17.50 ± 0.35^d^
30%	78.01 ± 0.04^b^	1.57 ± 0.03^de^	1.12 ± 0.05^c^	18.55 ± 0.20^b^
40%	78.79 ± 0.31^a^	1.62 ± 0.04^cd^	1.34 ± 0.04^a^	17.52 ± 0.36^d^
Two-way ANOVA				
Carbohydrate	0.0006	0.0001	0.0049	0.0004
variety	0.0074	0.0003	0.3359	0.0096
Carbohydrate × variety	0.6217	0.0004	0.2392	0.2037

*Note*: Data are means ± SD (*n* = 3). Values with different superscript letters in the same column show significant differences (*p*  < 0.05).

**Table 5:** Effect of diets with different carbohydrate levels on the amino acid composition of muscle in two varieties of carp.

Amino acid (mg/g)	YC			SSC			Two-way ANOVA		
	20%	30%	40%	20%	30%	40%	Carbohydrate	Variety	Carbohydrate × variety
Gly^#^	2.27 ± 0.04^a^	2.01 ± 0.09^b^	2.05 ± 0.01^b^	2.37 ± 0.09^a^	2.09 ± 0.01^b^	2.29 ± 0.06^a^	0.0057	0.003	0.4013
Ala^#^	2.55 ± 0.03^b^	2.66 ± 0.04^b^	2.88 ± 0.05^a^	2.9 ± 0.1^a^	2.92 ± 0.06^a^	2.87 ± 0.02^a^	0.0322	0.0886	0.0013
Ser	0.17 ± 0.01^c^	0.2 ± 0.01^bc^	0.2 ± 0.01^b^	0.2 ± 0.01^bc^	0.2 ± 0.01^bc^	0.25 ± 0.03^a^	0.0425	0.071	0.257
Pro	1.64 ± 0.02^d^	1.66 ± 0.02^cd^	1.74 ± 0.01^ab^	1.77 ± 0.03^a^	1.7 ± 0.02^bc^	1.76 ± 0.04^a^	0.0786	0.0004	0.0587
Val^*∗*^	1.44 ± 0.03^d^	1.61 ± 0.04^bc^	1.66 ± 0.02^ab^	1.61 ± 0.04^bc^	1.58 ± 0.03^c^	1.70 ± 0.05^a^	0.0113	0.0118	0.0699
Thr^*∗*^	1.64 ± 0.06^c^	1.83 ± 0.07^b^	2.01 ± 0.05^a^	1.98 ± 0.06^ab^	2.08 ± 0.06^a^	2.06 ± 0.13^a^	0.0301	0.0112	0.1767
Cys	0.57 ± 0.01^c^	0.77 ± 0.04^b^	0.59 ± 0.01^c^	0.73 ± 0.04^b^	0.59 ± 0.02^c^	0.89 ± 0.05^a^	0.004	0.004	0.0011
ILe^*∗*^	1.87 ± 0.05^c^	2.09 ± 0.04^b^	2.21 ± 0.03^a^	2.1 ± 0.03^b^	2.06 ± 0.04^b^	2.18 ± 0.05^a^	0.0032	0.0567	0.0386
Leu^*∗*^	3.26 ± 0.04^d^	3.61 ± 0.04^c^	3.8 ± 0.07^ab^	3.71 ± 0.06^bc^	3.62 ± 0.06^c^	3.8 ± 0.07^a^	0.0204	0.0162	0.0747
Asp^#^	4.4 ± 0.17^b^	3.99 ± 0.04^c^	5.03 ± 0.2^a^	5.13 ± 0.14^a^	4.49 ± 0.18^b^	4.44 ± 0.16^b^	0.0015	0.1961	0.0243
Lys^*∗*^	3.94 ± 0.04^d^	4.33 ± 0.09^c^	4.48 ± 0.1^bc^	4.58 ± 0.09^ab^	4.48 ± 0.12^bc^	4.6 ± 0.14^a^	0.0048	0.0035	0.1607
Glu^#^	5.17 ± 0.05^c^	5.58 ± 0.03^b^	5.67 ± 0.29^b^	5.84 ± 0.1^ab^	5.79 ± 0.08^ab^	6.03 ± 0.08^a^	0.02	0.0167	0.2426
Met^*∗*^	1.84 ± 0.04^d^	1.98 ± 0.02^c^	2.17 ± 0.01^b^	2.15 ± 0.02^b^	2.1 ± 0.08^b^	2.21 ± 0.05^a^	0.0078	0.0184	0.0333
His^&^	1.04 ± 0.02^c^	1.68 ± 0.06^a^	1.68 ± 0.06^a^	1.55 ± 0.06^b^	1.46 ± 0.04^b^	1.5 ± 0.06^b^	0.0042	0.2617	0.0004
Phe^*∗*^	2.35 ± 0.03^d^	2.8 ± 0.03^bc^	2.88 ± 0.04^ab^	2.77 ± 0.04^c^	2.76 ± 0.07^c^	2.90 ± 0.03^a^	0.0007	0.0134	0.0076
Arg^&^	2.07 ± 0.03^c^	2.2 ± 0.01^b^	2.32 ± 0.06^ab^	2.33 ± 0.07^a^	2.3 ± 0.06^ab^	2.35 ± 0.09^a^	0.0222	0.0893	0.1856
Tyr	1.5 ± 0.02^c^	1.66 ± 0.06^b^	1.7 ± 0.06^b^	1.71 ± 0.08^b^	1.64 ± 0.03^b^	1.82 ± 0^a^	0.0231	0.187	0.0367
Trp	0.14 ± 0^c^	0.18 ± 0.01^a^	0.16 ± 0.01^b^	0.15 ± 0^b^	0.15 ± 0.01^b^	0.18 ± 0^a^	0.0089	0.0539	0.01
Asn	2.11 ± 0.02^c^	2.32 ± 0.09^b^	2.7 ± 0.06^a^	2.61 ± 0.06^a^	2.67 ± 0.06^a^	2.65 ± 0.04^a^	0.0089	0.0182	0.0002
Gln	0.03 ± 0^d^	0.04 ± 0^c^	0.05 ± 0^b^	0.06 ± 0^a^	0.03 ± 0^d^	0.06 ± 0^a^	0	0.0261	0.0005
EAA	16.33 ± 0.28^d^	18.25 ± 0.22^c^	19.2 ± 0.25^b^	18.9 ± 0.4^b^	18.66 ± 0.41^bc^	19.45 ± 0.62^a^	0.0024	0.0018	0.0497
SEAA	3.11 ± 0.02^c^	3.89 ± 0.07^ab^	4.01 ± 0.15^a^	3.88 ± 0.15^ab^	3.76 ± 0.12^b^	3.86 ± 0.14^ab^	0.0083	0.1037	0.0054
NEAA	20.55 ± 0.32^c^	21.08 ± 0.11^c^	22.77 ± 0.62^ab^	23.47 ± 0.63^a^	22.26 ± 0.5^b^	23.24 ± 0.51^a^	0.0048	0.0226	0.0672
DAA	18.23 ± 0.36^c^	18.71 ± 0.16^c^	20.2 ± 0.69^ab^	20.72 ± 0.64^a^	19.69 ± 0.48^b^	20.44 ± 0.39^ab^	0.0095	0.0402	0.0821
TAA	39.99 ± 0.56^d^	43.21 ± 0.36^c^	45.97 ± 1.01^ab^	46.25 ± 1.17^a^	44.69 ± 1.01^bc^	46.52 ± 1.26^a^	0.0045	0.0126	0.046
EAA/TAA	40.84 ± 0.32^c^	42.23 ± 0.16^a^	41.77 ± 0.38^b^	40.86 ± 0.22^c^	41.77 ± 0.14^b^	41.7 ± 0.22^b^	0.0004	0.8196	0.0035
EAA/NEAA	79.49 ± 1.1^d^	86.57 ± 0.62^a^	84.35 ± 1.22^bc^	80.52 ± 0.56^d^	83.83 ± 0.43^c^	83.72 ± 0.95^c^	0.0004	0.8795	0.002

*Note*: Data are means ± SD (*n* = 3). Effects of diets with different carbohydrate levels on the amino acid composition of muscle in two varieties of carp. Values with different superscript letters in the same column show significant differences (*p*  < 0.05).

Abbreviations: DAA, total delicious amino acids; EAA, total essential amino acids; NEAA, total nonessential amino acids; SEAA, total semi-essential amino acids; TAA, total amino acids

*⁣* ^*∗*^Essential amino acids.

^#^Delicious amino acids.

^&^Semi-essential amino acids.

**Table 6:** Effect of diets with different carbohydrate levels on the fatty acid composition of muscle of two varieties of carp.

Fatty acid (%)	YC			SSC			Two-way ANOVA		
	20%	30%	40%	20%	30%	40%	Carbohydrate	Variety	Carbohydrate × variety
C14 : 0	1.58 ± 0.01^d^	1.69 ± 0.03^b^	1.64 ± 0.01^c^	1.5 ± 0^e^	1.63 ± 0.01^c^	1.72 ± 0.01^a^	0.000	0.123	0.000
C15 : 0	0.35 ± 0^b^	0.39 ± 0^a^	0.35 ± 0.01^b^	0.32 ± 0.01^c^	0.29 ± 0^d^	0.35 ± 0.01^b^	0.089	0.007	0.001
C16 : 0	26.42 ± 0.06^c^	29.67 ± 0.04^a^	29.7 ± 0.05^a^	27.73 ± 0.06^b^	25.04 ± 0.08^d^	26.23 ± 0.07^e^	0.000	0.000	0.000
C17 : 0	0.17 ± 0.01^b^	0.15 ± 0^c^	0.13 ± 0^e^	0.15 ± 0^d^	0.2 ± 0.01^a^	0.19 ± 0^a^	0.000	0.003	0.000
C18 : 0	9.35 ± 0.05^d^	11.7 ± 0.01^a^	11.14 ± 0.06^b^	10.48 ± 0.02^c^	7.1 ± 0.03^f^	7.94 ± 0.04^e^	0.001	0.032	0.000
C20 : 0	0.2 ± 0.02^ab^	0.2 ± 0.02^ab^	0.22 ± 0.01^a^	0.18 ± 0.01^c^	0.14 ± 0^d^	0.18 ± 0.01^bc^	0.000	0.000	0.000
C22 : 0	0.09 ± 0^a^	0.06 ± 0^c^	0.08 ± 0^b^	0.06 ± 0.01^c^	0.06 ± 0^c^	0.08 ± 0.01^b^	0.000	0.000	0.000
Total saturated fatty acids	38.17 ± 0.1^d^	43.87 ± 0.03^a^	43.26 ± 0.03^a^	36.7 ± 0.04^e^	34.45 ± 0.08^f^	40.42 ± 0.05^c^	0.000	0.001	0.024
C16 : 1	6.6 ± 0.03^c^	6.46 ± 0.02^d^	6.74 ± 0.02^b^	5.76 ± 0.02^f^	6.83 ± 0.03^a^	6.27 ± 0.02^e^	0.004	0.002	0.271
C20 : 1	1.27 ± 0.04^d^	1.23 ± 0.04^d^	1.15 ± 0.03^e^	1.43 ± 0.08^c^	1.85 ± 0.02^a^	1.69 ± 0.02^b^	0.000	0.001	0.000
C24 : 1	0.04 ± 0^c^	0.05 ± 0^c^	0.06 ± 0^b^	0.07 ± 0.01^a^	0.05 ± 0^c^	0.06 ± 0^ab^	0.034	0.001	0.232
C18 : 1n9c	35.92 ± 0.08^c^	31.82 ± 0.08^f^	33.18 ± 0.06^e^	34.71 ± 0.07^d^	40.37 ± 0.09^a^	38.04 ± 0.09^b^	0.001	0.005	0.003
C22 : 1n9	0.13 ± 0.01^b^	0.14 ± 0^a^	0.12 ± 0.01^c^	0.11 ± 0^d^	0.09 ± 0.01^f^	0.1 ± 0.01^e^	0.066	0.011	0.003
Total monounsaturated fatty acids	43.98 ± 0.05^c^	39.7 ± 0.08^f^	41.25 ± 0.05^e^	42.08 ± 0.08^d^	49.19 ± 0.09^a^	46.16 ± 0.05^b^	0.004	0.005	0.007
C18 : 2n6c	12.75 ± 0.04^b^	11.46 ± 0.06^e^	10.96 ± 0.04^f^	13.22 ± 0.01^a^	12.32 ± 0.16^c^	11.99 ± 0^d^	0.002	0.007	0.002
C18 : 3n3	1.48 ± 0.01^a^	1.14 ± 0.05^b^	1.1 ± 0.01^b^	0.97 ± 0.02^c^	0.97 ± 0.02^c^	0.95 ± 0.03^c^	0.001	0.015	0.009
C18 : 3n6	0.1 ± 0.01^d^	0.09 ± 0^e^	0.11 ± 0^bc^	0.11 ± 0^b^	0.1 ± 0^cd^	0.13 ± 0.01^a^	0.000	0.006	0.000
C20 : 4n6	0.34 ± 0.01^c^	0.33 ± 0.01^cd^	0.31 ± 0.01^d^	0.38 ± 0.01^b^	0.36 ± 0.03^b^	0.48 ± 0^a^	0.001	0.005	0.051
C20 : 2	0.16 ± 0^b^	0.12 ± 0.01^c^	0.12 ± 0.01^c^	0.17 ± 0.01^b^	0.17 ± 0.01^b^	0.19 ± 0.01^a^	0.000	0.007	0.000
EPA (C20 : 5n3)	0.36 ± 0.01^a^	0.32 ± 0^b^	0.31 ± 0.01^b^	0.37 ± 0.03^a^	0.26 ± 0.01^c^	0.24 ± 0.01^d^	0.000	0.000	0.000
DHA (C22 : 6n3)	0.77 ± 0.01^a^	0.62 ± 0^c^	0.53 ± 0.01^d^	0.59 ± 0.01^d^	0.46 ± 0.02^e^	0.43 ± 0.01^f^	0.000	0.000	0.000
Total polyunsaturated fatty acids	15.95 ± 0.06^a^	14.09 ± 0.11^e^	13.45 ± 0.04^f^	15.51 ± 0.02^b^	14.89 ± 0.01^c^	14.65 ± 0.15^d^	0.000	0.004	0.001

*Note*: Data are means ± SD (*n* = 3). Values with different superscript letters in the same column show significant differences (*p*  < 0.05).

Abbreviations: DHA, docosahexaenoic acid; EPA, eicosapentaenoic acid.

## Data Availability

Data will be made available upon request.

## Supporting Information

Supporting Information 1 Table S1: relative mRNA expression levels of glucose metabolism-related genes in the livers of carp fed the test diets.

Supporting Information 2 Table S2: the relative mRNA expression levels of genes involved in the synthesis of polyunsaturated fatty acids in the muscles of fish-fed experimental diets.
