# Supplementary material for: Effects of Different Carbohydrate Levels in Diets on Growth Performance and Muscle Nutritive Value of Ying Carp and Scattered-Scaled Mirror Carp (Cyprinus carpio)
Source: Aquac Nutr. 2025 Jan 28;2025:9966429. doi: 10.1155/anu/9966429 (PMC11824835; doi:10.1155/anu/9966429)
Supplement: Supporting Information 1 — Table S1: relative mRNA expression levels of glucose metabolism-related genes in the livers of carp fed the test diets. [file 9966429.f1.docx]

Relative mRNA expression levels of glucose metabolism-related genes in the livers of carp fed the test diets.

| *gene* | YC | | | SSC | | |
| --- | --- | --- | --- | --- | --- | --- |
|  | 20% | 30% | 40% | 20% | 30% | 40% |
| *glut2* | 1.39±0.08 | 6.07±0.81 | 2.28±0.33 | 2.71±0.19 | 4.38±0.62 | 2.86±0.53 |
| *pk1* | 1.32±0.22 | 1.56±0.08 | 3.48±0.81 | 2.44±0.39 | 3.92±0.13 | 9.27±1.38 |
| *pfk* | 0.89±0.11 | 1.01±0.14 | 0.24±0.02 | 0.95±0.13 | 1.09±0.16 | 0.97±0.06 |
| *g6p* | 4.64±0.28 | 4.43±0.22 | 1.75±0.18 | 1.1±0.15 | 2.49±0.31 | 1.13±0.18 |
| *pepck* | 1±0.07 | 0.13±0.03 | 0.61±0.06 | 0.52±0.02 | 0.73±0.12 | 0.5±0.11 |
| *fbp* | 1.01±0.15 | 3.25±0.23 | 0.72±0.14 | 1±0.08 | 0.65±0.06 | 1.4±0.12 |

*Note*: Data are means ± SD (n = 3).
