# Supplementary material for: Effects of Different Carbohydrate Levels in Diets on Growth Performance and Muscle Nutritive Value of Ying Carp and Scattered-Scaled Mirror Carp (Cyprinus carpio)
Source: Aquac Nutr. 2025 Jan 28;2025:9966429. doi: 10.1155/anu/9966429 (PMC11824835; doi:10.1155/anu/9966429)
Supplement: Supporting Information 2 — Table S2: the relative mRNA expression levels of genes involved in the synthesis of polyunsaturated fatty acids in the muscles of fish-fed experimental diets. [file 9966429.f2.docx]

| *gene* | YC | | | SSC | | |
| --- | --- | --- | --- | --- | --- | --- |
|  | 20% | 30% | 40% | 20% | 30% | 40% |
| *acsbg2* | 1.05±0.31 | 0.33±0.11 | 0.21±0.09 | 1.3±0.15 | 0.36±0.12 | 0.14±0.01 |
| *acat1* | 1.01±0.11 | 0.54±0.12 | 0.49±0.07 | 0.55±0.01 | 0.71±0.27 | 0.26±0.01 |
| *elovl5* | 1±0.07 | 0.81±0.2 | 0.57±0.06 | 0.57±0.05 | 0.27±0.06 | 0.24±0.03 |
| *lpl-α* | 1±0.03 | 0.44±0.03 | 0.58±0.09 | 0.58±0.04 | 0.46±0.08 | 0.3±0.08 |
| *elovl6* | 1±0.03 | 0.7±0.04 | 0.69±0.15 | 0.9±0.02 | 0.28±0.04 | 0.2±0.02 |
| *rxrgb* | 1±0.04 | 0.71±0.09 | 0.71±0.06 | 0.48±0.08 | 0.43±0.09 | 0.18±0.05 |
| *fabp3* | 1.02±0.21 | 1.1±0.25 | 1.09±0.09 | 1.88±0.41 | 1.14±0.29 | 1.14±0.36 |

The relative mRNA expression levels of genes involved in the synthesis of polyunsaturated fatty acids in the muscles of fish fed experimental diets.

*Note*: Data are means ± SD (n = 3)
